# Variable Range Hopping Model Based on Gaussian Disordered Organic Semiconductor for Seebeck Effect in Thermoelectric Device

**DOI:** 10.3390/mi13050707

**Published:** 2022-04-29

**Authors:** Ying Zhao, Jiawei Wang

**Affiliations:** 1 Key Laboratory of Microelectronic Devices & Integrated Technology, Institute of Microelectronics of Chinese Academy of Sciences, Beijing 100029, China; zhaoying@ime.ac.cn; 2 University of Chinese Academy of Sciences, Beijing 100049, China

**Keywords:** Seebeck coefficient, thermoelectric device, variable range hopping

## Abstract

We investigate the carrier concentration dependent Seebeck coefficient in Gaussian disordered organic semiconductors (GD-OSs) for thermoelectric device applications. Based on the variable-range hopping (VRH) theory, a general model predicting the Seebeck effect is developed to reveal the thermoelectric properties in GD-OSs. The proposed model could interpret the experimental data on carrier concentration- and temperature-dependence of the Seebeck coefficient, including various kinds of conducting polymer film and small molecule based field-effect transistors (FETs). Compared with the conventional Mott’s VRH and mobility edge model, our model has a much better description of the relationship between the Seebeck coefficient and conductivity. The model could deepen our insight into charge transport in organic semiconductors and provide instructions for the optimization of thermoelectric device performance in a disordered system.

## 1. Introduction

Thermoelectric materials and devices are of great interest in the pursuit to overcome the dilemma of global energy, in which heat can be directly transported or used to generate electricity based on the Peltier and Seebeck effects [1,2]. Thermoelectric efficiency is dominated by a dimensionless figure-of-merit, *ZT* = *S*^2^*σT*/*κ*, where *S*, *σ*, *T*, and *κ* are the Seebeck coefficient, electrical conductivity, absolute temperature, and thermal conductivity, respectively [1,2]. Owing to low thermal conductivity, potential low cost, and ease of low-temperature processing, organic semiconductors (Oss) are now considered promising thermoelectric candidates [3,4]. However, the development of Oss in thermoelectric generators is impeded by their low carrier concentration and poor electrical conductivity. Caused by the much weaker inter-molecular bonding for the van der Waals interaction strength (usually in the order of 100 meV) [5,6], Oss exhibit poor crystallinity, a low degree of intermolecular electronic coupling, and a high disorder degree, which severely impact the electrical performance. Even though the chemical doping can efficiently provide high electric performance of Oss, it also increases the structural and energetic disorder and broadens the density of states (DOS) [3]. The complicated and highly disordered OS system requires insight into more detailed information about charge transport mechanism and thermoelectric property. To further explore the potential of Oss in thermoelectric applications, it is indispensable to have a specific and certified transport model for thermoelectric characterization. However, there is still controversy about the transport models of Oss for predicting Seebeck effect.

Generally, Mott’s variable range hopping (VRH) and mobility edge (ME) models have been considered to describe phenomenological behavior of the charge transport in disordered OSs for decades [7,8]. Ideally derived from a constant DOS, for Mott’s VRH model, localized carrier tends to hop to a localized site that is far away but has a small energy difference; for Mott’s ME model, the localized carriers are completely immobile and transport via releasing onto the mobility edge with the aid of electric fields or enough thermal activation, whereas the classical VRH and ME models only provide limited assistance to studies on charge transport and thermoelectric property in amorphous OSs, due to the density of states (DOS) always being energy-dependent. In addition, it is suggested that Mott’s VRH and ME model might not capture the organic semiconductors’ Seebeck effects well [9].

In this work, we carry out a theoretic investigation on thermoelectric properties of gaussian disordered organic semiconductors (GD-OSs). Instead of Mott’s mode with constant DOS, we propose a model to describe the Seebeck effect with Gaussian density of states (DOS) and VRH theory, in which the contribution of energetic level to the conductivity and thermoelectric characters is clarified. It is proven that our VRH model could very well describe the observed dependence of Seebeck coefficients on carrier concentration, temperature, and conductivities, as long as the proper DOS is selected instead of a constant density at the Fermi level. Additionally, priority of our VRH model over Mott’s VRH and ME models is discussed, in that the shift of transport level makes the organic semiconductors have a larger Seebeck coefficient, even at very high carrier concentration. We believe the model is instructive for future thermoelectric related research, and that it interprets pre-existing experimental data on the Seebeck effect in disordered organic semiconductors.

## 2. Model Methods

In the practical disordered OSs, due to the complicated situation, the distribution of DOS is energy-dependent and inhomogeneous. If spatial positions and energies of the localized energy states can be considered independent from each other and random distribution of those states is described by a Gaussian energy spectrum, the OSs are so-called GD-OSs. Thus, a Gaussian DOS is assumed here for the energy-dependent distribution of localized sites [10].

(1) $$g\left(\varepsilon^{\prime }\right)=\frac{N_{t}}{\sqrt{2\pi }\delta^{\prime }k_{B}T}exp\left(-\frac{\varepsilon^{2}}{2\delta^{\prime^{2}}}\right),$$

where *N_t_* is the concentration of localized states, $\delta^{\prime }=\delta /k_{B}T$, and δ is the width of the Gaussian distribution reflecting the disorder’s degree. The localized states form a discrete array of hopping sites, in which the carriers transport as incoherent hopping. The probability for carrier hopping from an occupied site *i* to an empty site *j* is given by [11]:

(2) $$P_{ij}\propto \exp\left(-2\alpha R_{ij}-\frac{\left(\varepsilon_{j}-\varepsilon_{i}\right)+\left|\varepsilon_{j}-\varepsilon_{i}\right|}{2k_{B}T}\right),$$

where *α* is the decay constant of the assumed hydrogen-like localized wave function, *R_ij_* is the hopping distance, *ε_i_* and *ε_j_* are the localized sites’ energy, *k_B_* is Boltzman constant, and *T* is the temperature. For physical and mathematical simplicity, the Apsley’ hopping space method is employed later to make the energy space and real space equivalent, by defining the reduced coordinates $R^{\prime }=2\alpha R$ [12,13], $\varepsilon^{\prime }=\frac{\varepsilon }{k_{B}T}$, and the hopping range in the hopping space $R=R_{ij}^{\prime }+\left(\varepsilon_{j}^{\prime }-\varepsilon_{i}^{\prime }+/\varepsilon_{j}^{\prime }-\varepsilon_{i}^{\prime }/\right)/2$.

**Figure 1:**
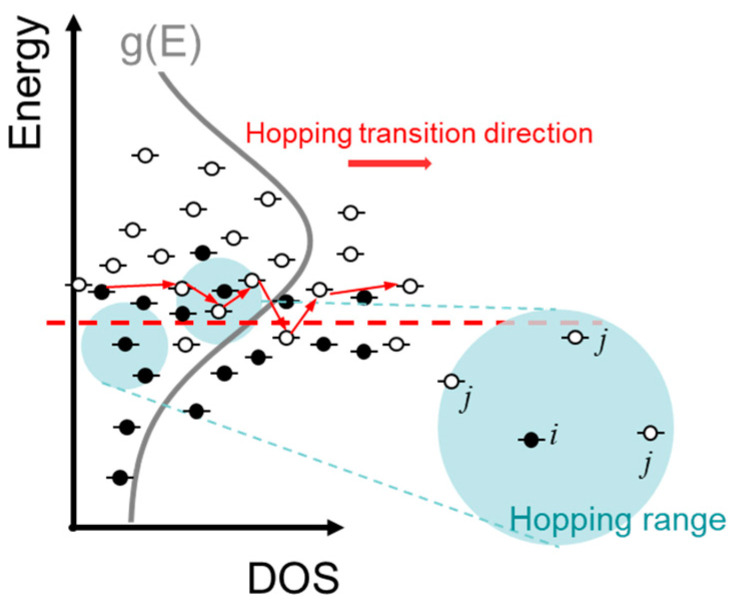
The schematic of carriers’ hopping near the transport energy. The blue region means the possible hopping range of carriers.

Generally in GD-OSs, the carriers tend to hop to a localized site with less energy difference and longer spatial distance. Thus, an essential condition for the formation of conduction paths is to find a certain number of empty sites within a certain energy and spatial range. Considering the inhomogeneous energy distribution of DOS, as shown in Figure 1, the adequate empty sites in the hopping range around site *i* causes percolation. The number of the empty sites in a range *R* respective to site *i* is determined by

(3) $$N\left(\varepsilon^{\prime },T,\beta ,R\right)=\frac{k_{B}T}{8\alpha^{3}}\int_{0}^{R}4\pi R^{\prime^{2}}dR^{\prime }\int_{-\infty }^{\varepsilon_{i}^{\prime }+R-R^{\prime }}g\left(\varepsilon_{j}^{\prime }\right)\left[1-f\left(\varepsilon_{j}^{\prime },\varepsilon_{f}^{\prime }\right)\right]d\varepsilon_{j}^{\prime },$$

where $f\left(\varepsilon_{j}^{\prime },\varepsilon_{f}^{\prime }\right)$ is the Fermi–Dirac distribution and $1-f\left(\varepsilon_{j}^{\prime },\varepsilon_{f}^{\prime }\right)$ represents the distribution of empty sites. The relationship between reduced Fermi level $\varepsilon_{f}^{\prime }=E_{f}/k_{B}T$ and carrier concentration n is given by $n=\frac{N_{t}}{\sqrt{2\pi }\sigma^{\prime }}\int_{-\infty }^{+\infty }d\varepsilon^{\prime }\frac{exp\left(-\varepsilon^{\prime^{2}}/2\delta^{\prime }\right)}{1+exp\left(\varepsilon^{\prime }-\varepsilon_{f}^{\prime }\right)}$, based on percolation theory with meliorated VRH model. The average hopping range $\bar{R}$ can also be obtained by setting the percolation criterion $N\left(\varepsilon^{\prime },T,\beta ,R\right)=2.8$ in Equation (3) in the situation of thick film (3-dimensional) [14]. Subsequently, the hopping diffusion constant is given by $D\left(\varepsilon_{i}^{\prime }\right)=\frac{R{\left(\varepsilon_{i}^{\prime }\right)}^{2}}{6{\left(2\alpha \right)}^{2}}v_{0}exp\left[-R\left(\varepsilon_{i}^{\prime }\right)\right]$, where *v*_0_ is the attempt-to-escape frequency, and the effective carrier mobility is obtained as $\mu =\frac{\int_{-\infty }^{+\infty }\sigma \left(\varepsilon_{i}^{\prime }\right)d\varepsilon_{i}^{\prime }}{nq}$, with $\sigma \left(\varepsilon_{i}^{\prime }\right)$ as the conductivity distribution function $\sigma \left(\varepsilon_{i}^{\prime }\right)=\frac{e^{2}}{k_{B}T}D\left(\varepsilon_{i}^{\prime }\right)f\left(\varepsilon_{i}^{\prime },\varepsilon_{f}^{\prime }\right)g\left(\varepsilon_{i}^{\prime }\right)$. The transport energy level *ε_t_* is defined as the energetic position of the most probably conductance $\sigma_{\max}$ occurs.

Finally, based on Fritzsche’s model $S=-\frac{1}{q}\int \left(\frac{E-E_{f}}{k_{B}T}\right)\frac{\sigma \left(E\right)}{\sigma }dE$ [15,16], the Seebeck coefficient is expressed as

(4) $$S=\frac{\left[\int \left(\varepsilon_{i}^{\prime },\varepsilon_{f}^{\prime }\right)\sigma \left(\varepsilon_{i}^{\prime }\right)d\varepsilon_{i}^{\prime }\right]}{qT\left[\int \sigma \left(\varepsilon_{i}^{\prime }\right)d\varepsilon_{i}^{\prime }\right]},$$

denoting the energy carried by the electrons (holes) per unit charge, where the energy is measured with respect to the Fermi level. Based on the developed physical model, the influence of relative physical and experimental parameters on charge transport and thermoelectric property can be related and analyzed. For instance, different doping concentrations bring increases in both carrier concentration *n* and disorder’s degree δ [17], while the percolation criterion $N\left(\varepsilon^{\prime },T,\beta ,R\right)$ can be adjusted to match different situations of charge transport under different film dimensionality.

## 3. Results and Discussion

**Figure 2:**
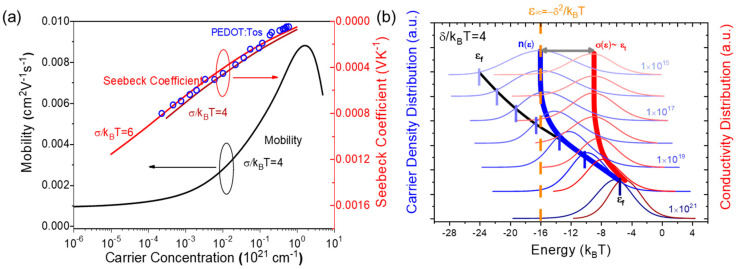
(**a**) Carrier concentration of mobility (black line) and Seebeck coefficient (red lines) in Gaussian disordered organic semiconductor, with fitting data from PEDOT:Tos film (blue circles). (**b**) Schematic of the energy position evolution with carrier concentration for carrier density distribution (blue lines) and conductivity distribution (red lines); orange dashed line indicates the position of equilibrium level, blue dashed line stands for the “main source” from which the carriers are activated, and red dashed lines indicate the transport energy level.

Figure 2a shows the carrier concentration *n*-dependent mobility and Seebeck coefficient, the parameters are *N_t_* = 1 × 10^22^ cm^−3^, *α* = 2 × 10^7^ cm^−1^, *T* = 300 K, and *v*_0_ = 1 × 10^13^ s^−1^. It is observed that the mobility (with Gaussian width *δ* = 4 *k_B_T*) exhibits strong *n*-dependent properties when carrier concentration is larger than around *n* = 1 × 10^18^ cm^−3^, and gradually becomes *n*-independent, reflecting carriers’ saturated diving process in DOS tail. The result is consistent with Blom’s and Baranovski’s theoretic interpretations of OCC-PPV and P3HT’s data [18,19]. The Seebeck coefficient *S* increases with the carrier concentration, decreasing when the carrier concentration is high, and is weakly dependent on the degree of disorder. When the carriers concentration drops around *n* = 1 × 10^18^ cm^−3^, mobility becomes *n*-independent, while the Seebeck coefficient keeps monotonically decreasing and exhibits *n*-dependent properties over the whole range of carrier concentration. Physically, the saturation of mobility at low carrier concentration arises from the invariable activation energy *E_a_* that carriers need during the process of hopping from equilibrium level *ε_∞_* to transport level *ε_t_* [20]. However, the Seebeck coefficient is directly linked to the energy that carriers bring, which is defined relative to the Fermi level *ε-ε_f_*, having no relation with the equilibrium level *ε_∞_.*

As Figure 2b shows, the most probable distribution position of carriers could be identified in the vicinity of *ε_f_* at higher carrier concentration while gradually stabilizing at *ε_∞_* in spite of the downward shifting Fermi level. Simultaneously, the distribution of conductivity or the transport energy *ε_t_* at various carrier concentration is plotted. Different to mobility edge *ε_c_*, transport energy *ε_t_* would rise gradually with an increase in carrier concentration *n,* especially at larger values of *n*. Unlike the drastic fall in the Seebeck coefficient at higher conductivity in Mott’s ME model, our VRH model might predict a slower decrease in the Seebeck coefficient with increasing carriers’ concentration. Experimental data for doped PEDOT-Tos film [21] displayed as symbols in Figure 2a are well fitted by our theoretic model, with input parameters *N_t_* = 1 × 10^22^ cm^−3^, *α* = 2 × 10^7^ cm^−1^, *v*_0_ = 1 × 10^13^ s^−1^, and *δ* = 6 *k_B_T*.

**Figure 3:**
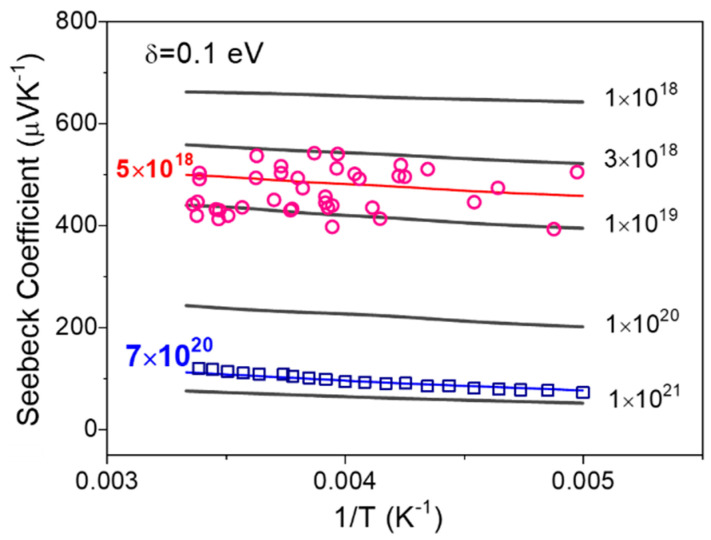
Temperature dependence of Seebeck coefficient at Gaussian width *δ* = 0.1 eV; experiment data for fitting are from pentacene based FET (red circles), and polymer film based on polyacetylene (blue square).

The proposed model also predicts the temperature’s effect on the Seebeck coefficient at different carrier concentrations in Figure 3, in which the input parameters are *N_t_* = 1 × 10^22^ cm^−3^, *α* = 2 × 10^7^ cm^−1^, *v*_0_ = 1 × 10^13^ s^−1^, and *δ* = 0.1 eV. At higher carrier concentration, the Seebeck coefficient is predicted to slowly increase with temperature, consistent with the polyacetylene experimental results for carrier concentration *n* = 7 × 10^20^ cm^−3^, shown in Figure 3 [22,23,24]. In the moderate carrier concentration region, at room temperature and below, the Seebeck coefficient also exhibits weak temperature dependence, consistent with experimental data of a pentacene-based organic FET, in which the fitting parameters of carrier concentration in our model coincides with that actual accumulated in an operated OFET (*n*~10^18^~10^19^ cm^−3^).

**Figure 4:**
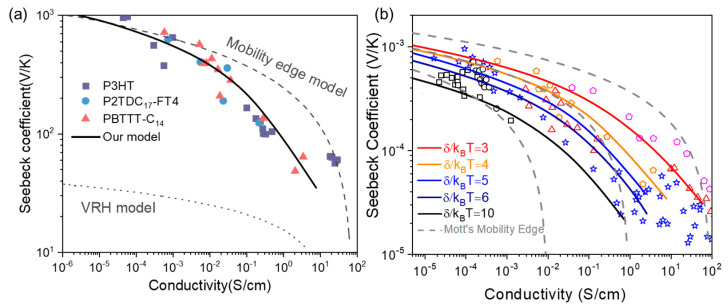
Relationship between conductivity and Seebeck coefficient: (**a**) comparison of fitting results between our model and Mott’s VRH/ME model including P3HT, P2TDC_17_-FT4, and PBTTT-C_14_; (**b**) fitting between theory in this work and experimental data given by the literature, including P3HT:F4TCNQ mixed P3HTT film (black squares and black circles), polyacetylene (blue stars), PBTTT film (orange pentagons and red triangles), and PEDOT:PSS (magenta pentagons).

The relationship between conductivity and the Seebeck coefficient is instructive for the design and optimization thermoelectric devices, as the general Seebeck coefficient’s variation trend with conductivity follows a regular pattern. In Figure 4a, the proposed model could well fit several experiments’ data from the literature [9,25], while Mott’s ME and VRH models cannot match them at all. The deviation of Mott’s VRH from the conductivity–Seebeck coefficient relationship should be attributed to the assumption of constant density of states near the Fermi level, *N*(*E*_f_) = const. Considering our model, hopping does not necessarily occur near the Fermi level. The energetic position *ε_t_* of the most probable conductance varies with temperature and carrier concentration; therefore, the density of states is not a constant parameter. As long as a proper DOS is employed together with reasonable hopping parameters, it could ideally model the conductivity–Seebeck coefficient relationship with our VRH model using Apsley’ hopping space method (as Equations (2)–(4) shown).

The experimental data from P3HT, PBTTT, polyacetylene, and PEDOT:PSS are plotted in Figure 4b [26,27,28,29], and the solid lines are drawn with our hopping model. We enclosed one set of experimental data with our fit lines’ envelope by selecting rational input parameter of disorder degree *δ*. As an example of P3HT data (black squares and black circles in Figure 4b), the inter-lines transition from one with larger Gaussian width *δ* at higher conductivity to that of a lower, less doped one could be identified. The input parameters are *N_t_* = 1 × 10^22^ cm^−3^, *α* = 2 × 10^7^ cm^−1^, and *v*_0_ = 1 × 10^13^ s^−1^ for *δ* = 4, 5, 6 and 10 *k_B_T*, and *v*_0_ = 3 × 10^13^ s^−1^ for *δ* = 3 and 1.5 *k_B_T*. The apparent deviation of Mott’s ME model from the experiments can be compared by dashed lines ($s=-\frac{1}{qT}\left[\left(E_{C}-E_{f}\right)+A_{c}\right]$, $\sigma =\sigma_{ME}exp\left[-\left(E_{C}-E_{f}\right)/k_{B}T\right]$) in Figure 4b, where the σ_ME_ is metallic conductivity at mobility edge *ε_c_*, by setting different values σ_ME_ = 100, 1, and 0.01 S/cm. It is due to this that the invariance of mobility edge *ε_c_* makes the charge carried thermo-power decrease quickly with Fermi level (*ε_c_*-*ε_f_*) when carrier concentration increases. Meanwhile, in our VRH systems, the transport energy *ε_t_* rises together with *ε_f_*, making the thermo-power (*ε_t_*-*ε_f_*) decrease much more gently with the conductivity.

Via statistically analyzing the listed model parameters in the fitting results, we found that the charge transport and thermoelectric property of different kinds of doped OSs can be well-reproduced by only adjusting the degree of disorder *δ* and the attempt-to-escape frequency *v*_0_. This suggests that the doped organic conducting materials are totally disordered systems, better depicted by hopping limited transport than by multiple trapping and release transport in Mott’s ME system. The evolution of transport energy *ε_t_* is essential for the thermo-power at higher carrier concentrations, which could not be expected when the transport is dominated by ME. For achieving a higher Seebeck coefficient, together with higher conductivity in thermoelectric devices, the hopping-limited transport model featured by transport energy *ε_t_*, together with lower energetic disorder, is expected. This needs more systematic investigations in the future, for the two factors are not easy realized simultaneously.

## 4. Conclusions

In summary, based on variable range hopping theory and Apsley’s hopping space method, we present a model based on the Seebeck effect to reveal the thermoelectric properties in a disordered organic semiconductor with Gaussian DOS. The proposed model could interpret the experimental data on a carrier concentration- and temperature-dependent Seebeck coefficient including doped polymer and small molecule-based FETs from the literature. Compared with Mott’s VRH and ME models with constant DOS, the relationship between the Seebeck coefficient and conductivity could be better fitted by our model. This model could provide instructive references and guidance for future research on thermoelectric characteristics of disordered organic systems.
